# A tissue attached to self-expandable metal stents for biliary stricture could be useful to find malignancy

**DOI:** 10.1038/s41598-022-24115-7

**Published:** 2022-11-16

**Authors:** Hyungjun Kwon, Min Kyu Jung, Sung Jin Park, Jun Heo, Dong Wook Lee, Yoo Na Kang, Jaryung Han, Young Seok Han, Won Kee Lee

**Affiliations:** 1 Department of Surgery, Kyungpook National University School of Medicine, Daegu, South Korea; 2 Department of Internal Medicine, Kyungpook National University School of Medicine, 680 Gukchaebosang-Ro, Jung-Gu, Daegu, 41944 South Korea; 3 Division of Gastroenterology and Hepatology, Department of Internal Medicine, Kyungpook National University Hospital, Daegu, South Korea; 4 Department of Forensic Medicine, Kyungpook National University School of Medicine, Daegu, South Korea; 5 Department of Surgery, Kyungpook National University Hospital, Daegu, South Korea; 6 Department of Surgery, Daegu Catholic, University School of Medicine, Daegu, South Korea; 7 Department of Medical Informatics, Kyungpook National University School of Medicine, Daegu, South Korea

**Keywords:** Bile duct cancer, Bile ducts

## Abstract

Biliary strictures can have several benign or malignant causes. We attempted to determine the usefulness of establishing a diagnosis using self-expandable metal stents (SEMS) in a prospective series of patients with suspected malignant biliary obstruction. Data of patients who underwent SEMS removal from August 2016 to December 2019 were collected. During this period, 55 patients underwent endobiliary biopsy and SEMS insertion and removal. Fifty-five consecutive patients (mean age, 69 years; range 53–90 years) were enrolled, and of these, 37 were male and 18 were female. A final diagnosis was established using biopsy specimens in 37 cases (67.3%) and surgical specimens in 6 cases (10.9%), with 12 cases (21.8%) diagnosed on radiological follow-up. The final diagnoses included malignancy in 34 cases (61.8%) and benign stricture in 21 cases (38.2%). Endobiliary biopsy had a sensitivity and specificity of 44.1% and 95.2%, whereas SEMS cytology had a sensitivity and specificity of 52.9% and 100%, respectively. Combining endobiliary biopsy and/or SEMS cytology yielded a sensitivity and specificity of 73.5% and 95.2%, respectively. (1) The use of biopsy results alone as a diagnostic tool yielded an area under the receiver operating characteristic curve (AUC) of 0.70 (0.60–0.79). (2) The addition of SEMS to the biopsy results yielded an AUC of 0.86 (0.78–0.94). (3) The addition of CA 19–9 levels to the biopsy results yielded an AUC of 0.81 (0.71–0.94). (4) Combining the endobiliary biopsy results, SEMS tissues, and CA 19–9 levels yielded the best diagnostic accuracy, with an AUC of 0.90 (0.83–0.98). Detection of biliary obstruction using the combination strategy was better than the diagnostic results based on biopsy alone according to recent 3-year data. Our study suggested that SEMS removal could help establish a diagnosis of suspected malignant biliary obstruction.

## Introduction

It is challenging to make a correct diagnosis of the biliary stricture. The Biliary strictures can generally be classified as benign or malignant. Benign biliary strictures are typically caused by choledocholithiasis, but other causes are post cholecystectomy strictures, inflammation, stricture formation secondary to pancreatitis and idiopathic causes, choledochal cysts, primary sclerosing cholangitis, and Mirizzi syndrome. On the contrary, malignant biliary obstruction is most commonly caused by cholangiocarcinoma, but other causes are carcinoma of the gall bladder, carcinoma of the head of pancreas, enlarged lymph nodes, and metastasis^1^.

Bile duct cancer has been commonly pathologically diagnosed using tissue sampling obtained by brush cytology and/or biliary biopsy forceps performed during endoscopic retrograde cholangiopancreatography (ERCP)^2,3^. Although ERCP remains the mainstay and primary method of tissue diagnosis, the diagnostic yield from such a procedure is low. During ERCP, bile samples may be collected to assess the presence of cancer cells. Biopsy specimens can also be obtained during cholangioscopy, which allows physicians to evaluate the inside surface of the bile duct and obtain samples from suspicious areas^4^. However, reports have shown a 6–72% sensitivity for malignancy with bile cytology and a 29–81% sensitivity with biliary biopsy^3,5–8^.

For malignant biliary obstruction, decompression via endoscopic stent placement can mitigate jaundice and pruritus. Endoscopic stent placement into the common bile duct is a relatively routine procedure, and technical success is achieved in over 90% of cases. Thus, this has become the most common method for achieving biliary decompression^9^. Over the past decade, the use of self-expandable metal stents (SEMS) for the treatment of malignant biliary strictures has become more common^10^. However, emerging studies have shown that SEMS occlusion now occurs in approximately half of all patients with malignant biliary strictures. Although removing the non-covered biliary metal stent has not been feasible in recent years, some studies have suggested that SEMS can be safely removed^11,12^.

We believe that the cellular components attached to the surface of the removed SEMS could be pathologically evaluated. The low sensitivity of traditional methods has led us to determine whether pathological diagnosis using removed SEMS is possible. As such, this study aimed to determine whether removed SEMS could help establish a diagnosis in cases with suspected malignant biliary obstruction.

## Methods

### Patients

Our prospectively collected database of SEMS removal procedures performed from August 2016 to December 2019 at Kyungpook National University Hospital in Daegu was analyzed. During this period, 55 patients underwent endobiliary biopsy and SEMS insertion and removal. Based on the clinical history and imaging studies, those who underwent SEMS placement at the stricture were suspected to have malignant biliary strictures. Patients with incomplete data were excluded from this study. This study was approved and the informed consent was waived by the Institutional Review Board of Kyungpook National University Hospital. All methods were performed in accordance with the Declaration of Helsinki.

All patients underwent endobiliary biopsy at least once, during which SEMS were placed for the biliary stricture. The SEMS were removed when the patient was treated for obstructive jaundice or acute cholangitis due to stent malfunction. The removed SEMS were prepared for cytology in the same manner as a tissue specimen. Demographic data, CA 19–9 levels, endobiliary biopsy and SEMS cytology results, and final diagnoses were determined by reviewing the medical records.

### ERCP procedures

All procedures were performed at a single academic referral center. ERCP was performed by a dedicated therapeutic endoscopist (M.K. Jung) who performs over 1000 ERCP procedures annually using standard techniques. Endobiliary biopsies with or without brush cytology were routinely performed in all cases. After endobiliary biopsy, the therapeutic endoscopist placed the SEMS to dilate the biliary stricture. Follow-up ERCP was then performed for SEMS removal, reevaluation, or exchange if malignancy was confirmed. The follow-up ERCP was usually performed 3 months later. Upon follow-up ERCP, the removed biliary stent was sent for cytology analysis.

### Preparation of SEMS for cytology

**Figure 1:**
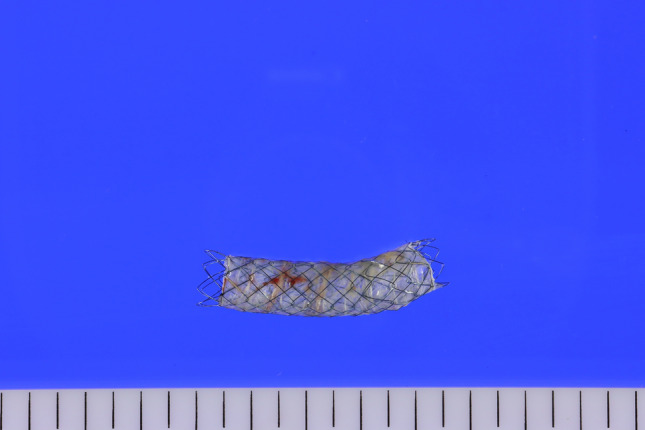
The removed self-expandable metal stents.

Upon arrival at the cytology laboratory, the stent was removed after which the attached material was rinsed into a fixative (CytoLyt, Hologic Inc., MA, USA). The tissue, attached to self-expandable metal stents, was spontaneously separated from the SEMS (Fig. 1). The sample was centrifuged at 2000 rpm for 10 min and affixed to the CytoLyt for 30 min. The supernatant was discarded, and the cell pellet was placed into the ThinPrep 5000 automated slide processor (Hologic Inc., MA, USA) for liquid-based cytology. A single ThinPrep monolayer cytology slide was prepared and stained using the Papanicolaou method. This cytology slide was come from the tissue from SEMS. So in this study I used the term “SEMS cytology” to clearly discriminate from bile cytology or brush cytology.

Diagnostic reporting of these specimens followed standard cytology categorization as follows: non-diagnostic (i.e. a cytology specimen that provides no diagnostic or useful information about the lesion sampled), negative for malignant cells, atypical (i.e. cytologic changes that are more likely than not to be benign), suspicious for malignant cells (i.e. cytologic changes that are more likely than not to be malignant), and positive for malignant cells. In this study, malignant disease was considered when the cytologic category was atypical, suspicious for malignant cells, or positive for malignant cells.

### Endobiliary biopsy

Two specimens were obtained from the biliary stricture during endobiliary biopsy. For biopsies diagnosed as category 1, which indicates negativity for neoplasia/dysplasia (including normal, reactive, regenerative, hyperplastic, atrophic and metaplastic epithelium), further follow-up of the lesion may or may not be necessary, as clinically indicated. In the case of category 2, which is indefinite for neoplasia/dysplasia, follow-up is needed due to uncertainty regarding the true nature of the lesion. For category 3, which indicates non-invasive low-grade neoplasia (low-grade adenoma/dysplasia), neoplasia is present, but the risk for developing invasive carcinoma is low. For category 4, which indicates non-invasive high-grade neoplasia, the risk for invasion and development of metastases is increased. In the case of category 5, which indicates invasive neoplasia, the risk of subsequent deeper invasion and metastases is so high that treatment is urgently needed and should only be withheld in cases with clinical contraindications. Generally, before a treatment is selected, the possibility of sampling errors should always be considered, which may cause underestimation of the grade of neoplastic change or depth of invasion. We followed the Vienna classification of gastrointestinal epithelial neoplasia^13^. In this study, malignant disease was considered when the biopsy showed category 3, 4, or 5 disease.

### Final diagnosis

The final diagnosis was established using (1) cytologic and/or histologic evidence obtained by tissue sampling during ERCP, endoscopic or percutaneous fine needle biopsy, surgery, or autopsy or (2) clinical data obtained during follow-up of at least a year. Definitely malignant and severely suspicious cytological or histopathological findings were classified as positive. Nearly all tissue samples acquired by brush cytology or forceps biopsy were examined by two local pathologists with appropriate expertise. When brush cytology and forceps biopsy were performed during ERCP, tissue samples obtained by both modalities were examined by the same pathologists. A stricture was considered benign when no evidence of malignancy was observed after follow-up for at least 1 year (i.e. absence of radiological tumor progression including infiltration and/or metastatic dissemination).

### Statistical analysis

Results were expressed as means ± standard deviations or as percentages. Statistical analysis was performed using the chi-square test, Student’s *t *test, or Fisher’s exact test. Test characteristics for endobiliary biopsy, SEMS cytology, and endobiliary biopsy with SEMS cytology, including sensitivity, specificity, positive predictive value (PPV), and negative predictive value (NPV) were calculated. Moreover, we compared receiver operating characteristic curve values for (1) biopsy, (2) biopsy and SEMS cytology, (3) biopsy and CA 19–9, and (4) biopsy, CA 19–9, and SEMS cytology. All statistical analyses were performed with SAS 9.4 (SAS Institute Inc., Cary, NC, USA), and p values < 0.05 indicated statistical significance.

## Results

Fifty-five consecutive patients (mean age, 69 years; range 53–90 years) were enrolled in this study, among which 37 were male and 18 were female. The diameter of the SEMS was 8 mm in 9 patients (16.4%), 10 mm in 44 patients (80%), and 12 mm in two patients (3.6%). The demographic data and clinical features of the 55 analyzed patients are summarized in Table 1.

**Table 1:** Demographic and clinical features of the analyzed population.

Characteristics	Values
Age (year, mean ± SD)	69.1 ± 15.0
Males/females	37/18
**Metal stent diameter**	
8 mm	9 (16.4%)
10 mm	44 (80%)
12 mm	2 (3.6%)
**Final diagnosis**	
Malignancy	34 (61.8%)
CBD cancer	17
Klatskin tumor	4
Pancreatic cancer	13
Benign	21 (38.2%)
Benign biliary stricture	12
Chronic pancreatitis	4
IgG4-related sclerosing cholangitis	4
Mirizzi syndrome	1

*CBD* common bile duct, *SD* standard deviation.

The final diagnosis was established using biopsy specimens in 37 cases (67.2%) and surgical specimens in six cases (10.9%), while 12 cases (21.8%) were diagnosed on radiological follow-up. The final diagnosis was malignancy in 34 patients (61.8%) and benign stricture in 21 patients (38.2%). The causes of biliary stricture are described in Table 1. The causes of malignant biliary strictures included common bile duct cancer, pancreatic cancer, and Klatskin tumor. The causes of benign strictures included unknown benign stricture, chronic pancreatitis, IgG4-related sclerosing cholangitis, and Mirizzi syndrome. The basic characteristics of benign and malignant biliary strictures are described in Table 2. Patients in the malignant biliary stricture group had higher CA 19–9 levels than those in the benign group (p = 0.03).

**Table 2:** Basic characteristics of patients with benign and malignant biliary strictures.

Basic characteristics	Benign	Malignant	p value
Age (year, mean ± SD)	65.4 ± 11.8	71.4 ± 16.7	0.152
Males/females	19/2	18/16	0.010
**CA 19–9**			
< 37 IU/L	11	7	0.032
≥ 37 IU/L	10	27	
**CRP**			
< 0.5 mg/dL	12	11	0.126
≥ 0.5 mg/dL	9	23	
**Bilirubin**			
< 1.2 mg/dL	10	8	0.012
≥ 1.2 mg/dL	11	26	

*SD* standard deviation.

Diagnostic accuracies, sensitivities, specificities, PPVs, and NPVs are detailed in Table 3. Endobiliary biopsy had a sensitivity and specificity of 44.1% and 95.2%, whereas SEMS cytology had a sensitivity and specificity of 52.9% and 100%, respectively. Combining endobiliary biopsy and/or SEMS cytology yielded a sensitivity and specificity of 73.5% and 95.2%, respectively. The combined strategy had a better sensitivity than endobiliary biopsy alone (p = 0.014).

**Table 3:** Sensitivity, specificity, and accuracy rates of the diagnostic methods.

	Biopsy	Biopsy + SEMS Cx	p values	SEMS Cx	Biopsy + SEMS Cx	p values
	(n = 55)	(n = 55)		(n = 55)	(n = 55)	
True positive (n)	15	25		18	25	
True negative (n)	20	20		21	20	
False positive (n)	1	1		0	1	
False negative (n)	19	9		16	9	
Sensitivity	44.1% (15/34)	73.5% (25/34)	0.014	52.9% (18/34)	73.5% (25/34)	0.078
Specificity	95.2% (20/21)	95.2% (20/21)	1.000	100.0% (21/21)	95.2% (20/21)	0.312
Positive predictive value	93.8% (15/16)	96.2% (25/26)	0.722	100.0% (18/18)	96.2% (25/26)	0.400
Negative predictive value	51.3% (20/39)	69.0% (20/29)	0.143	56.8% (21/37)	69.0% (20/29)	0.310
Accuracy	63.6% (36/54)	81.8% (45/55)	0.070	70.9% (45/54)	81.8% (45/55)	0.835
AUC (95% CI)	0.697 (0.6000–0.794)	0.844 (0.755–0.932)		0.765 (0.680–0.850)	0.844 (0.755–0.932)	

*AUC* area under the curve, *CI* confidence interval, *SEMS Cx* self-expendable covered metal stent cytology.

**Figure 2:**
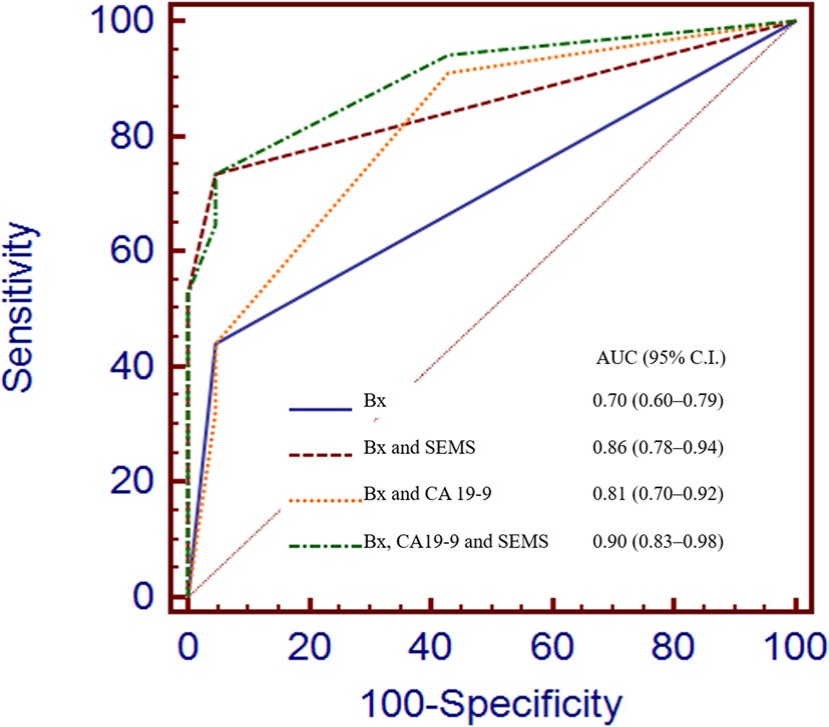
Receiver operating characteristic curves of each diagnostic method. *AUC* area under the curve, *Bx* endobiliary biopsy, *C.I*. confidence interval, *SEMS* self-expandable metal stent cytology.

Tissues obtained from the removed SEMS in addition to those obtained by endobiliary biopsy could help establish a more accurate diagnosis of biliary strictures (Fig. 2). (1) The use of biopsy alone as a diagnostic tool yielded an area under the curve (AUC) of 0.70 (0.60–0.79). (2) The addition of SEMS to the biopsy result yielded an AUC of 0.86 (0.78–0.94). (3) Addition of the CA 19–9 level to the biopsy results yielded an AUC of 0.81 (0.71–0.94). (4) The use of endobiliary biopsy, SEMS tissue cytology, and CA 19–9 levels yielded the best diagnostic accuracy, with an AUC of 0.90 (0.83–0.98).

## Discussion

A diagnosis of biliary strictures can significantly alter treatment management plans due to the need for aggressive treatment of confirmed malignancies and avoiding unnecessary surgery in benign conditions^14^. ERCP with standard forceps biopsy and/or brush cytology has often been the first-line diagnostic approach^2,3^. However, this approach is limited by its low sensitivity and high rates of false-negative results. A systematic review and meta-analysis (9 studies, 730 patients) of the effectiveness of ERCP for detecting malignant biliary strictures showed that the pooled sensitivities of forceps biopsy and brush cytology were 45% and 48.1%, respectively^14^. In the current study, endobiliary biopsy had a sensitivity of 44.1%.

In contrast, in several studies, potentially valuable samples were discarded after a stent was retrieved from a patient whose diagnosis remained uncertain^12,14,15^. Such studies have reported sensitivities ranging from 11 to 78%, as some had small sample sizes^12,14,15^. A recent studied reported a diagnostic yield of 1.6% for any malignancy despite that most of the stents were composed of plastic^16^. Evidence has shown that the SEMS can decompress and relieve biliary strictures in patients with indeterminate biliary strictures^9,10^. Although SEMS can be safely removed, it is impossible to remove the uncovered biliary metal stent^11,12^. Nonetheless, upon SEMS replacement or removal, subsequent SEMS cytology may be a potential method for the pathologic diagnosis of biliary strictures. The current study showed that SEMS cytology had a sensitivity of 52.9% and an NPV of 56.8%, which implies that a negative result was sufficient to rule out cancer.

The current study suggested that the combination of endobiliary biopsy and/or SEMS cytology is better than when biopsy is used alone. When pathology and/or radiology is unable to provide information regarding malignancy, SEMS cytology may be utilized to obtain pathologic information. For example, our findings showed a sensitivity and specificity of 73.5% and 95.2%, respectively. Moreover, the current study showed that a combination strategy had better sensitivity than endobiliary biopsy alone (p = 0.014).

Recently, a single-operator peroral cholangioscopy (SOC) system with four-way deflected steering and separate working and irrigation channels has become available and has addressed some of the shortcomings of previously used instruments (SpyGlass direct visualization system; Boston Scientific Corp., MA, USA.)^17^. According to experiences published by tertiary centers in the US and Europe, SOC appears to be useful for the diagnosis of indeterminate biliary lesions and has an accuracy of 80–89% for differentiating malignant and benign lesions^18–21^. However, complete visual evaluation of the biliary mucosa and obtaining biopsy samples from indeterminate biliary lesions are quite challenging. Moreover, the SpyGlass system is costly, as our estimates show an additional cost of $3000 per patient. Any information obtained before using the SpyGlass system would be pertinent and impactful.

This study has several limitations worth noting. First, the number of patients was small, which limits the power and avoidance of a type II error. Second, although we included all patients who underwent SEMS removal with endobiliary biopsy, selection bias is possible given that this study was retrospective. Further large-scale prospective studies are necessary to overcome these limitations.

In conclusion, several highly sensitive and specific diagnostic options, such as cholangioscopy and endoscopic ultrasound-guided fine needle aspiration or biopsy, are currently available for pancreaticobiliary malignancies. However, these procedures are not only expensive but also technically demanding. Despite the remaining controversies surrounding SEMS cytology, pathologic examination of the SEMS, rather than discarding it, could be substantially useful.

The current study suggests that the combination of endobiliary biopsy and SEMS cytology is better than biopsy alone. As such, when no information regarding malignancy can be obtained from pathology and/or radiology, SEMS cytology could be used to obtain pathologic information.

## Data Availability

The datasets used and analyzed in this study are available from the corresponding author upon reasonable request.

## Abbreviations

AUC: Area under curve
CBD: Common bile duct
ERCP: Endoscopic retrograde cholangiopancreatography
NPV: Negative predictive value
PPV: Positive predictive value
ROC: Receiver operating characteristic curve
SEMS: Self-expandable metal stents
SOC: Single-operator peroral cholangioscopy
